# The pH-Dependent Stucture and Properties of Au and Ag Nanoparticles Produced by Tryptophan Reduction

**DOI:** 10.1186/s11671-016-1318-8

**Published:** 2016-02-24

**Authors:** Iuliia Mukha, Nadiia Vityuk, Olga Severynovska, Anna Eremenko, Nataliia Smirnova

**Affiliations:** Chuiko Institute of Surface Chemistry, National Academy of Sciences of Ukraine, 17, General Naumov Str., Kyiv, 03164 Ukraine

**Keywords:** Gold, Silver, Nanoparticle, Tryptophan, pH, UV/visible, Mass spectrometry, 78.67.Bf (Nanocrystals, nanoparticles, and nanoclusters), 33.15.Ta (Mass spectra), 87.15.Fh (Bonding; mechanisms of bond breakage)

## Abstract

In the work, an attempt was made to combine different experimental conditions to obtain stable gold and silver nanoparticles in the presence of amino acid tryptophan. The pH-dependent properties of gold and silver nanoparticles were studied. UV/visible spectroscopy and laser desorption/ionization mass spectrometry data confirm kynurenine pathway for tryptophan conversion in such systems.

## Background

Nanoparticle-based medicine has a huge potential for use in cancer diagnosis, detection, imaging, and treatment. Among others, the therapeutic direction for noble metal nanoparticles (NPs), especially in the field of cancer, is rapidly developing [1–5]. The authors have recently reported cytotoxic effect of mono- and bimetallic gold and silver NPs, stabilized with commonly used surfactants [6]. And the bimetallic NPs (gold/silver combination) exhibited the highest cytotoxic activity towards primary tumor cells and were the less harmful for hepatocytes and lymphocytes. The data collected in animal models suggested strong antitumor activity of obtained nanosized metals. To reduce potential hepato- and nephrotoxicity, an essential amino acid tryptophan (Trp) was used in nanoparticle synthesis as reducing/stabilizing agent [7]. The use of animal models confirmed effectiveness of NPs in inhibition of primary tumor growth: the rate of development of metastatic lesions was lowered and life expectancy increased.

To obtain stable bimetallic gold/silver nanoparticles with maximal antitumor effect, some experimental conditions must be optimized especially pH of the medium as it has an influence on the initial state of the reagents. Different metal hydroxo complexes formed at higher pH combined with amino acid in cationic/anionic form can strongly affect the mechanism of reaction and stabilization of the nanosized metal. So, the paper is focused on pH-dependent properties of gold and silver nanoparticles produced by tryptophan reduction with the main attention paid to mechanism of amino acid conversion.

## Methods

Colloidal solutions of monometallic silver and gold NPs were obtained via chemical reduction of aqueous solution of silver nitrate and tetrachlorauratic acid (AgNO_3_, HAuCl_4_, Merck, Germany) with amino acid tryptophan (Trp, SC12-20120713, China). The components interacted in a molar ratio *ν*(M):*ν*(Trp) = 1:1. The concentrations in the resulting solution used were C(M) = 10^−4^ M.

The absorption spectra of the colloidal solutions of Ag(Au) NPs were recorded in the UV/visible region with a spectrophotometer, Lambda 35 (PerkinElmer, USA), in 1-cm quartz cells.

The particle size distribution function and zeta potential measurement were studied by a laser correlation spectrometer Zeta Sizer Nano S (Malvern, UK) equipped with a correlator (multi-computing correlator type 7032 ce) by method based on the scattering of light on any micro-objects. The information signal from the random movement of nanoparticles was analyzed by multi-channel spectrum analyzer and colorimeters. One millimeter of the studied suspension was placed in a cylindrical optical glass cell with a diameter of 10 mm, which was located in a thermostatted sample holder of a laser correlation spectrometer. Registration and statistical processing of the scattered laser light at 173° from the suspension (helium-neon laser LGN–111 was used with power output of 25 mW and wavelength of 633 nm) were performed three times during 120 s at 25 °C. The resulting autocorrelation function was treated with standard computer programs PCS–Size mode v 1.61.

The pH measurements were performed using a pH-meter I-160MI. As a working electrode, a glass electrode was used. Silver chloride electrode served as a reference electrode. The acidity of the solution was varied by adding nitric acid and sodium hydroxide.

Mass spectrometry analysis was performed by the method of laser desorption/ionization (LDI MS) on an Autoflex II (Bruker Daltonics, Germany) mass spectrometer with nitrogen laser (*λ* = 337 nm). Experiments were carried out in reflectron mode for positive and negative ions in the mass range from 30 to 1000 Da. Resulting mass spectra were obtained by assuming the data of 200 laser shots and processing by the software FlexAnalysis (Bruker Daltonics, Germany).

## Results and Discussion

### Gold Nanoparticles

In aqueous solution of tetrachlorauratic acid, the rearrangement of coordination sphere of metal occurs at different pH. The hydroxo complexes [AuCl_4−x_(OH)_x_]^−^ are formed that play a key role in the process of formation of nanosized metal, especially in the case of such a weak reductant as tryptophan. In [8], the equilibrium forms of [AuCl_4_]^−^, [Au(OH)_3_Cl]^**−**^, and [Au(OH)_4_]^**−**^ at pH = 2, 6, and 11 were determined. During the reduction process, these hydroxo complexes serve as sites for nucleation and subsequent growth of nanoparticles [9].

Amino acid is also transformed with the change of pH. According to dissociation constants, tryptophan can exist in cationic, neutral, and anionic forms at рН 2, 6, and 10, respectively, and thus affect the reduction/stabilization process.

Zero approach for gold revealed that metal reduction is more favorable in the acidic medium (that was achieved by adding hydrochloric acid) confirmed by the corresponding  remaining plasmon absorption band, while at high pH, hydroxo complexes were almost not reduced and Au colloids almost were not formed. This process was accompanied by dramatic change in the absorption spectra.

Focusing on obtaining the final bimetallic AgAu particles, we changed some experimental conditions and expanded the pH range for gold (pH = 2, 4, and 6). Also, the use of nitric acid for control of acidity decreased the influence of chlorine ions on the process of silver reduction excluding the formation of low soluble chloride.

**Fig. 1:**
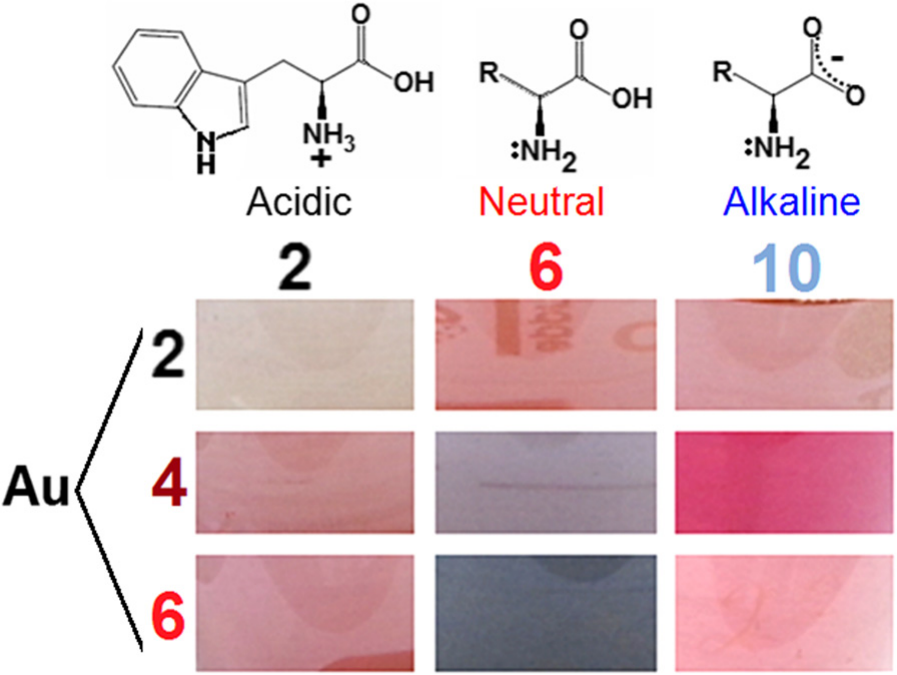
The color of gold colloids obtained from initial reagents used at different pH. Tryptophan was used in cationic (acidic medium, рН = 2), neutral (neutral medium, рН = 6), and anionic (alkaline medium, рН = 10) forms. HAuCl_4_ had initial pH = 2, 4, and 6

**Fig. 2:**
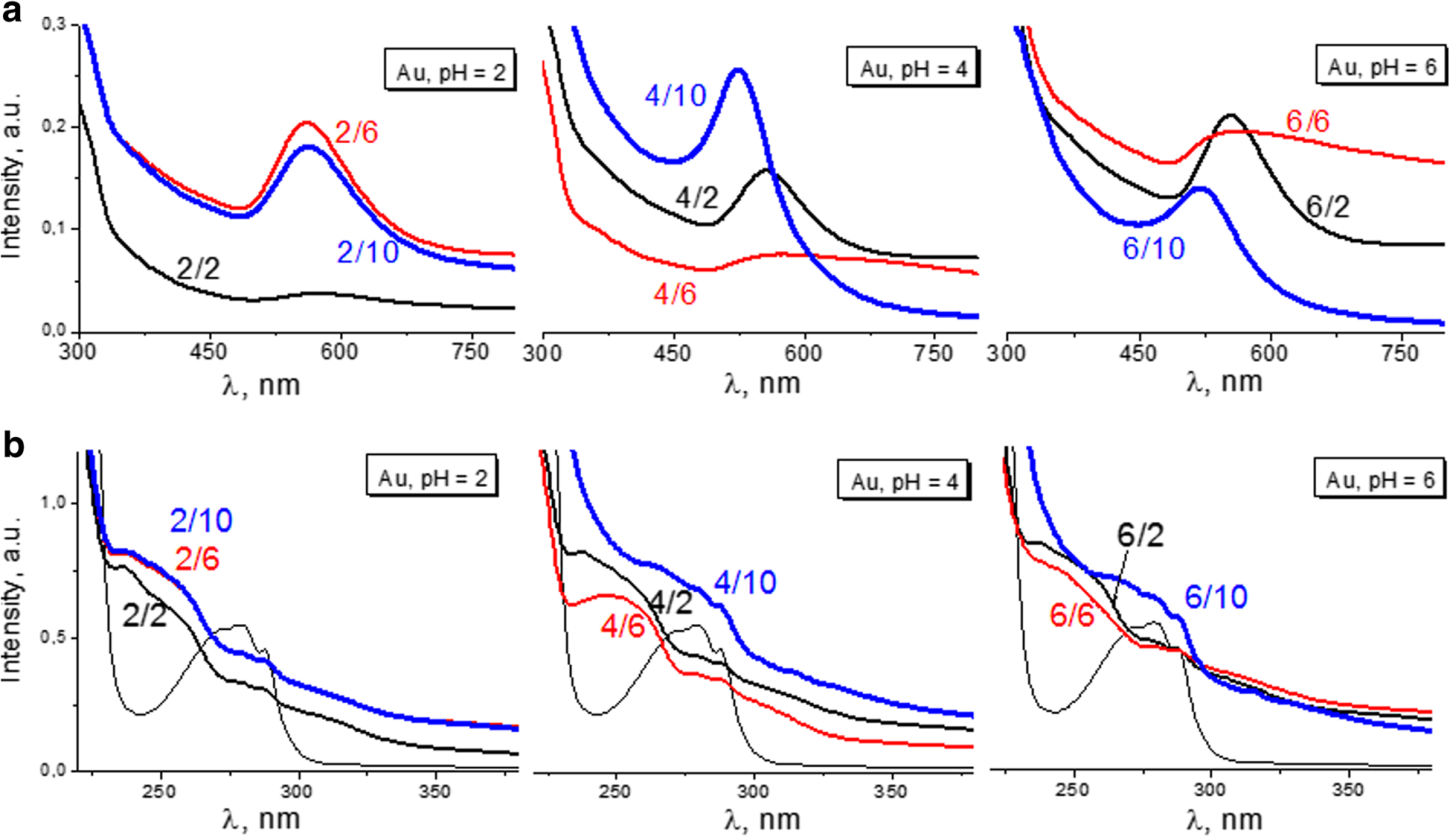
Optical spectra of gold colloids. **a** SPR absorption region. **b** Tryptophan absorption region. “a/b” refers to “initial pH of HAuCl_4_/initial pH of Trp”

Color and absorption profiles do not look similar for NPs obtained from solutions with different combinations of pH of initial reagents (Figs. 1 and 2a). Typical metal surface plasmon resonance (SPR) bands were present in optical spectra of colloids with maxima around 520–570 nm.

Fast reduction of gold occurred in the acidic medium. One of the parameters that determines the participation of amino acids in the reduction process is the ionization potential of the molecule, which for tryptophan in aqueous solution is 4.45 eV [10] and is provided by the presence of π-electrons. Tryptophan is quite sensitive to autooxidation [11]. Aromatic amino acids, especially tryptophan, have a high tendency to oxidation directly on metal electrodes due to interaction with the π-system of the indole molecule [12, 13]. One molecule of tryptophan could lose four electrons by direct oxidation [14] that is enough to reduce Au^3+^ до Au^0^.

Almost all gold colloids had aggregated particles (Table 1). For solutions with low resulting pH, they were of 100–150 nm. In particular, system “4/6” and “6/6” aggregates were larger and the color of solutions got blue with a drastic change in colloidal stability accompanied by disappearance of the SPR band (Fig. 2b).

**Table 1:** The pH-dependent properties of metal nanoparticles produced by tryptophan reduction

pH Trp→	2	6	10
pH Au ↓	Size, nm		
2	70–90/100–130	50–70/100–130	60–80/100–130
4	50–70/100–130	90–130/> 600	10–25
6	20–30/100–130	30–40/200–400	10–20
	Zeta potential, mV		
2	+15.2	+19.25	+24.5
4	+13.5	−5.27	−11.85
6	−0.03	−3.03	−0.04
	pH (resulting solution)		
2	2.2	2.5	2.5
4	2.5	3.9	7.5
6	2.5	6.4	7.6

Opposed to acidic medium, the reduction of gold took a long time when Trp was used at high pH. In the case of anionic Trp, stabilization is due to the donor-acceptor bonding that involves *d*-shell of the metal and electron density of deprotonated carboxylic group -COO^−^ and also nonbonding pair of electrons on the nitrogen atom of the amino group. The strong binding of other amino acids arginine and aspartate as functionalizing agents for gold NPs due to their unprotonated amine groups at high pH was also observed by Zare et al. [15]. Joshi et al. [16] based on theoretical calculations offered formation of metal–molecule hybrid orbitals, including *d-*orbital of gold and molecular orbitals of Trp, i.e., the amino, carboxyl groups, and indole ring, having a mixed character.

We determined the optimal system “4/10,” when alkaline amino acid and Au at pH = 4 were used, that resulted in the smallest and the most stable Au NPs having the most pronounced and symmetric SPR band with maximum at 520 nm. Also considering a pH of 7.5 of resulting colloids, this system was chosen for further investigation, aiming to obtain stable bimetallic AgAu nanoparticles.

The zeta potential of gold nanoparticles after capping with tryptophan is shown in Table 1. The surface of gold nanoparticles (“4/10”) carries a negative charge. Particle aggregation is less likely to occur for charged particle due to electrostatic repulsions. Probably, not only the presence of adsorbed ions on the surface of NPs but also the oxidation products of tryptophan that create an ionic strength provide the stability of the solution. Taking into account the zeta potential and previous fluorescent measurements [17], we can suggest the formation of charge transfer complexes between metal and amino acid. Some molecules form the inner sphere of the gold (I) complex and some of them are involved in reduction process.

The metabolism of tryptophan can take place in two possible pathways: (1) to neurotransmitter serotonin and (2) niacin (through kynurenine formation). The question arises about possible oxidation products of tryptophan in Au/Trp system. the serotonin pathway keeps the indole ring in the molecule, and thus there are no significant alterations in the absorption profile. In contrast, the shape and position of the amino acid absorption bands of the obtained colloids changed. Characteristic maxima of such oxidation products as N-formylkynurenine (NFK) and kynurenine (Kyn) were not pronounced at 318 and 361 nm [18], but the shift of band maxima in the ultraviolet region indicated the C_2_-C_3_ bond breaking in indole ring (Fig. 2). As the reaction proceeds and gold nanoparticles are formed, the absorption wavelength of the π–π∗ transition [19] shifts from 279 nm (unreacted amino acid) to 247 and 237 nm, respectively. Selvakannan et al. [20] observed similar shift and considering NMR data supposed oxidative polymerization of the indole group of the tryptophan molecules during reduction of the gold ions.

To identify tryptophan conversion, mass spectra of Au/Trp systems and individual molecules of tryptophan under the same experimental conditions were obtained. It should be noted that individual molecules in these conditions were not ionized.

**Fig. 3:**
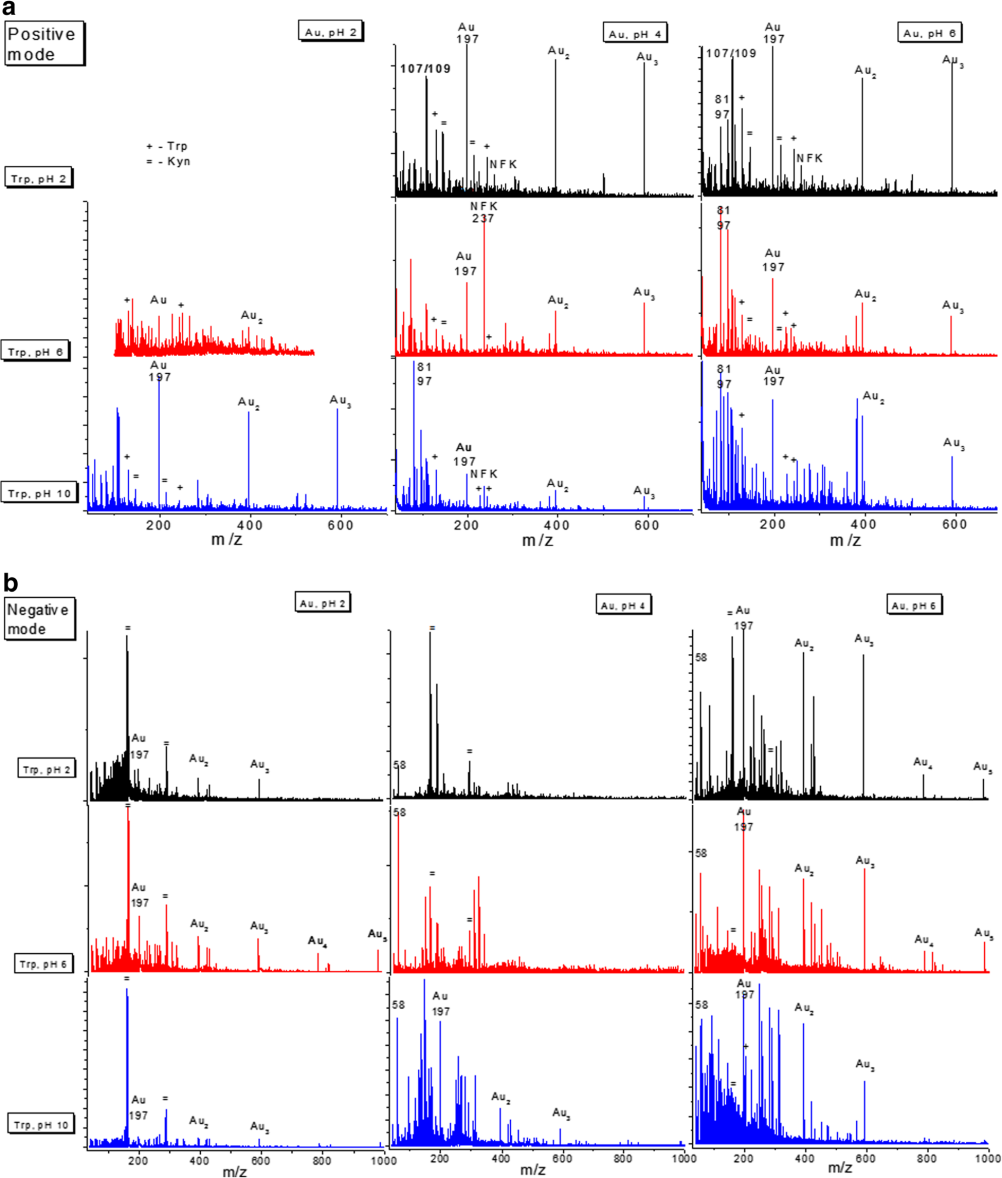
Representative fragment of mass spectra of gold colloids under consideration in mass range 40 to 1000 Da for positive (**a**) and negative (**b**) ions (reflectron). *Plus sign* corresponds to observed tryptophan fragments, *equal sign* corresponds to kynurenine fragments

For all systems in mass spectra for positive and negative ions, the series of intense single monoisotopic peaks with a pitch of 197 Da that refers to the average mass of Au were observed (Fig. 3). In mass spectra for positive ions, the series of less intense peaks in mass range from 108 m/z with the same pitch were registered. We suppose that a peak with the mass of about 108 Da belongs to unidentified Trp fragment, which formed associates with various amounts of Au atoms.

Individual tryptophan for all samples in the positive mode is characterized by the presence of peaks with 243 and 227 m/z that belong to fragments [Trp + K^+^]^+^ and [Trp + Na^+^]^+^ accordingly. Peak 130 m/z corresponds to ionized indole moiety C_9_H_8_N^+^ [21].

The difference in the character of mass spectra is determined by the resulting pH of samples (Table 1). The most pronounced tendency was observed for colloids with resulting acidic medium. For those systems, mass spectra were analyzed and interpreted in accordance with [22]. For positive ions, masses of 214 m/z [192 m/z + Na^+^]^+^ (where mass 192 Da refers to [[Kyn − NH_3_] + Na+]^+^) and 146 m/z, belonging to fragment [Kyn − NH_3_ − H_2_O − CO]^+^, were detected. We attribute them to the sequential kynurenine fragmentation.

Although two mechanisms of tryptophan conversion are possible, analysis of the data allows us to assume the preference of kynurenine pathway.

For the same samples in negative mode, we observed the combination of repeated intense peaks with masses about 162 m/z, referring to fragment [Kyn − H_2_O − CO]^−^, and about 290 m/z (possible belong to dimer of fragment with mass 146 Da (see above)). This fact is an additional confirmation of kynurenine pathway.

The presence of the peak with 203 m/z in the negative mode, corresponding to deprotonated molecule, is characteristic only for the limiting case—sample “6/10.” This sample presumably contains the maximum amount of unconverted molecules that is confirmed by absorption spectroscopy data (Fig. 2).

Colloidal “4/6” system had the slightly acidic medium with most aggregated particles and the maximum of absorption band at 247 nm. The peak with mass 237 m/z, which corresponds to fragment [NFK + H^+^]^+^, in mass spectra of this system was observed. This fact allows us to present N-formylkynurenine as a product of reaction.

In some cases, a low-intensity peak of this product at 259 m/z [NFK + Na^+^]^+^ was determined as intermediate in the oxidation process.

Peaks with masses 81 and 97 m/z in the positive mode and 58 m/z in the negative mode appeared in the systems with preformed hydroxo complexes especially for initial pH 6 for gold. These peaks supposedly correspond to associate non-aromatic moiety of tryptophan with Na and K. And also in mass spectra in negative mode peaks with masses about 313 and 429 m/z are present, which correspond to [Au(58)_2_]^−^ and [Au(58)_4_]^−^ complexes, respectively.

Thereby, in the acidic environment of Trp, more complete oxidation occurs and kynurenine residues were presented, in contrast to initial alkaline medium, when the spectra contained peaks of tryptophan. This tendency was observed in both ionization modes. Thus, from two possible ways of Trp conversion, the kynurenine pathway was shown as preferable.

Oxidation to kynurenine was more intense in acidic medium and accompanied with aggregation of gold nanoparticles. At the same time at high pH small NPs were formed and stabilized with Trp molecules.

The correlation between the data obtained by LDI MS and UV/vis spectroscopy suggests that the MS method may be applied to assess the mechanisms of redox reactions in Au Trp systems.

### Silver Nanoparticles

In the case of silver, the reduction of the metal occurs only in the presence of tryptophan in anionic form and also when silver exists at high pH. During the synthesis, the same approach was used when other conditions are equal to the Au NP synthesis. The rate of the redox reaction in this case is much slower than the rate of acid-base interaction, and therefore the synthesis of colloids required continuous heating.

**Fig. 4:**
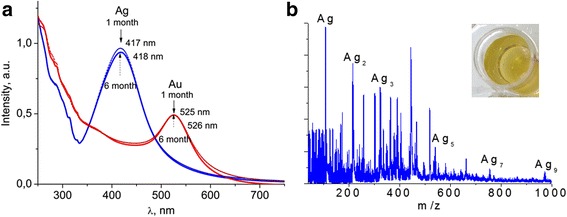
Optical spectra of silver and gold nanoparticles after 6 month of storing (**a**); mass spectra of Ag NPs under consideration in mass range 40 to 1000 Da for positive ions (**b**). The *inset* shows the color of silver colloid

Optical spectra of silver nanoparticle solutions with typical yellow color contained characteristic SPR bands at 417 nm (Fig. 4a). Repeated MS peaks with isotopic distributions indicated the presence of silver-cluster ions Ag_n_ and (Ag_n_ + 42 m/z) in both modes (Fig. 4b). According to LCS measurements, the average diameter of obtained NPs was 15–25 nm. What is really worth mentioning is the extreme stability of such silver colloids that is determined by the magnitude of zeta potential of nanoparticles, which is −27 mV and is confirmed by optical spectra, namely the SPR band position and intensity of its maximum. When colloidal solutions were stored during half a year at room temperature, the position of SPR maxima and their intensity for both metals remained almost unchanged. This is an evidence of extremely stable solution of metal nanoparticles (Fig. 4a).

After the redox process is finished, the pH of resultant colloidal solution of silver nanoparticles becomes neutral, that is due to the formation of acidic residues of Trp. Such pH allows the use of colloids in physiological environments.

The experimental conditions determined for obtaining stable monometallic gold and silver nanoparticles were used for further synthesis of bimetallic “alloy” AgAu nanoparticles.

## Conclusions

The formation and stabilization of gold and silver nanoparticles in the presence of tryptophan is strongly influenced by acidity of initial components. According to mass and absorption spectroscopy data, tryptophan conversion in such systems goes through the kynurenine pathway. The highest stability and lowest dispersity of nanometals occur in the case of metal reduction with amino acid in anionic state that exists in initial alkaline medium.

## Abbreviations

Kyn: kynurenine
LCS: laser correlation spectroscopy
LDI MS: laser desorption/ionization mass spectrometry
NFK: N-formylkynurenine
NPs: nanoparticles
SPR: surface plasmon resonance
Trp: tryptophan
